# The strange history of atmospheric oxygen

**DOI:** 10.14814/phy2.15214

**Published:** 2022-03-28

**Authors:** John B. West

**Affiliations:** ^1^ Department of Medicine University of California, San Diego La Jolla California USA

**Keywords:** *Cyanobacterium*, extinction, great oxidation event, oxygen generation, *Prochlorococcus*

## Abstract

Many of us think a lot about oxygen. This includes how the normal body handles oxygen in health, but particularly how this is complicated by lung disease. Few of us are aware that as human inhabitants of the earth, we have a unique privilege. This is that as air breathers, we and most other animals on Earth, are the only living creatures in the known universe that have unlimited supply of oxygen. This situation came about through one of the greatest miracles of nature, that is photosynthesis, the ability to release oxygen from water using the energy of sunlight. One consequence of this was that the first atmospheric oxygen came from the metabolism of microorganisms, the cyanobacteria, that used photosynthesis, but for which oxygen was an unwanted by‐product. In fact, the oxygen had to be discarded for the organisms to thrive. When a major increase of oxygen concentration in the atmosphere occurred some 2 billion years ago, and the partial pressure of oxygen in the air rose to perhaps 200 mmHg, this Great Oxidation Event as it was called, was a death sentence for the large population of anaerobic animals for whom oxygen was toxic. Today much of the oxygen in the atmosphere comes from photosynthesis in microorganisms, including the cyanobacteria, and the recently discovered *Prochlorococcus*, that discard this unwanted by‐product. The result is that the PO2 in our atmosphere at sea level remains nearly constant at about 150 mm Hg, although the factors responsible for this are not understood.

## Graphical abstract

Today much of the oxygen in the atmosphere comes from photosynthesis in microorganisms, including cyanobacteria and the recently discovered *Prochlorococcus* which is thought to be exceptionally efficient at harvesting light. The result is that the PO2 in our atmosphere at sea level remains nearly constant at about 150 mm Hg, although the factors responsible for this are not understood.

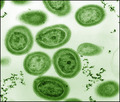

## 1 THE BEGINNINGS OF OXYGEN IN THE EARTH’S ATMOSPHERE

At the outset, it should be emphasized that many aspects of the origin and subsequent history of oxygen on our planet remain controversial, so this brief essay should be regarded as an introduction that hopefully will stimulate further interest in this fascinating topic.

When our solar system began to form some 4–5 billion years ago, it apparently consisted of a number of solid components with some accompanying gas. The system was held together by gravity. There was little or no free oxygen in the gas which is thought to have been a mixture of nitrogen, hydrogen, helium, methane, and some other elements of low atomic numbers. This situation continued over millions of years as our solar system with its eight planets (and Pluto) gradually evolved but without any significant amounts of atmospheric oxygen.

**FIGURE 1:**
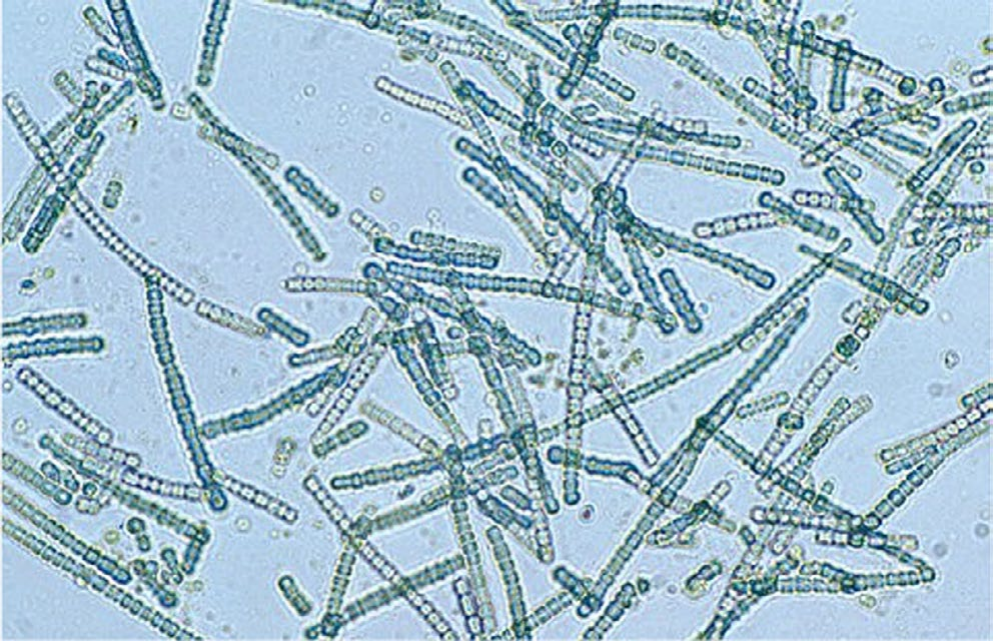
Cyanobacteria. These have a blue‐green color. They first appeared early in the evolution of the Earth’s atmosphere. About 2 billion years ago. They were responsible for the Great Oxidation Event as shown in Figure 2. From Wikipedia, in the public domain

Then an extraordinary change occurred. This was due to the miracle of photosynthesis which enabled some organisms to use the energy in sunlight to break water into its component molecules, oxygen and hydrogen. One consequence was that some gaseous oxygen began to appear. This ultimately resulted in a sea change for the future inhabitants of planet Earth as we see today. This initial oxygen was brought about by the emergence of a family of microorganisms called cyanobacteria (Figure 1). Cyano means blue, and these cells have a blue‐green coloring. The origin of these organisms is not known. However, these cells had the capability of photosynthesis that is they could conserve and exploit the energy from light. To do this they contained photosynthetic pigments related to chlorophyll which is well known because of its critical role in the metabolism of plants. In fact, the chlorophyll of plants that we see today is believed to have evolved from these pigments in cyanobacteria.

The cyanobacteria were capable of using carbon dioxide, and splitting water into its component molecules, hydrogen and oxygen. A typical equation is:

$$6{\mathrm{CO}}_{2}+\;6H_{2}O\;+\;\mathrm{energy}\;=\;C_{6}H_{12}O_{6}\;+\;6O_{2}.$$

The hydrogen was combined with carbon to make a variety of hydrocarbons, while the oxygen was eliminated as an unwanted byproduct. It is from this unlikely origin that the enormous families of oxygen‐dependent forms of life that we know today have developed including our own.

Where do we find the largest reservoir of oxygen in planet earth today? Since the atmosphere is composed of 21% oxygen, and this surrounds the whole of the globe up to an altitude of more than 80 km, it is natural to assume that most of the oxygen is located here. However, this is not so. By far the largest amount of oxygen by weight (over 90%) is in solid chemical forms such as oxides and silicates. In fact, if we add the amounts of oxygen in the atmosphere, the oceans, and in living creatures, the sum is less than 0.05% of the total mass of oxygen in planet earth (Knoll et al., 2012).

Some investigators believe that there have been fluctuations in the levels of atmospheric oxygen in more recent times. For example, one study proposes that vascular land plants caused a rise of oxygen in the Phanerozoic some 300 million years ago. These calculations suggest that the level may have increased to as much as 30% (Berner & Canfield, 1989).

What are the largest sources of oxygen in the world today? As described above, cyanobacteria and other photosynthetic phytoplankton continue to be major sources of oxygen. A recently described novel microorganism, the marine *Prochlorococcus*, is now recognized to be a major source and this is described below. However, there are other large sources too. One is marine sediments of organic matter where dead animals settle to the bottom of the ocean. Another is plate tectonics whereby shifts of the earth’s crust release oxygen from volcanoes and other sources in the earth’s mantle. Plants and trees also release significant amounts of oxygen. However, the importance of this source is sometimes exaggerated. For example, forest fires in the Amazon jungle are sometimes blamed for reducing atmospheric oxygen but the effect is very small.

## 2 THE GREAT OXIDATION EVENT

**FIGURE 2:**
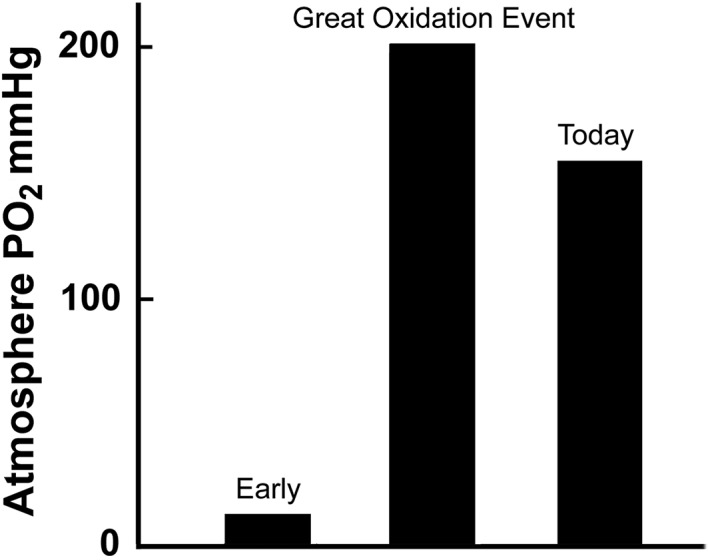
The dramatic increase in the atmospheric oxygen during the Great Oxidation Event. The oxygen level subsequently fell to that which we have today

AS indicated above, some oxygen was released by cyanobacteria early in the history of the earth. However, the atmospheric concentration of oxygen remained low for millions of years at the value of perhaps less than 10 mmHg. The reasons for this low value are not certain but it is suggested that it was because of absorption of the oxygen by the ocean and some land surfaces. But about 2 billion years ago in the Paleoproterozoic era, there was a dramatic increase in the atmospheric oxygen concentration (Figure 2.). As the figure shows, the partial pressure of oxygen increased from the extremely low value to around 150 mmHg. Although our planet has had more than its share of dramatic events such as ice ages, this gigantic change in the composition of the atmosphere was unprecedented.

This dramatic change in the environment had enormous consequences. For one thing, it helped to bring about the vast variety of oxygen‐dependent species that we see on the earth today. But there was also a big downside. The enormous increase in oxygen concentration had disastrous consequences for the many living species that were anaerobic. In fact, the ensuing extinction was one of the most disastrous biological events that our planet has ever known.

What were the reasons for this enormous rise in atmospheric oxygen? It is embarrassing to relate that in spite of an enormous amount of research and a corresponding avalanche of articles, we remain largely ignorant in this area. A typical article is titled “The continuing puzzle of the Great Oxidation Event” (Sessions et al., 2009) which discusses the presumed sources and sinks of oxygen that are presumably relevant, but it does not come to any convincing conclusions about the likely mechanisms. The article does discuss some of the markers for the presence or absence of oxygen. For example, it discusses Banded Iron Formations that are relatively common. These are red horizontal seams that are easily seen in rocky terrains, and are one of the best known relevant geologic markers. The article points out that these indicate anoxic conditions in the Archean epoch, but this information is of little value in explaining the mechanisms of the Great Oxidation Event (Olejarz et al., 2021).

## 3 RECENT DISCOVERY OF AN ENORMOUS SOURCE OF ATMOSPHERIC OXYGEN

The present article covers some 4–5 billion years of history, and it therefore seems anomalous to emphasize events that have occurred in the last 50 years. But it is a remarkable fact that within this time, discoveries have been made that may revolutionize our understanding of oxygen in the present atmosphere. Of course, details of such recent important discoveries will no doubt be modified over the next 50 years, but it is stimulating to discuss them here, bearing in mind that changes in thinking will undoubtedly occur.

**FIGURE 3:**
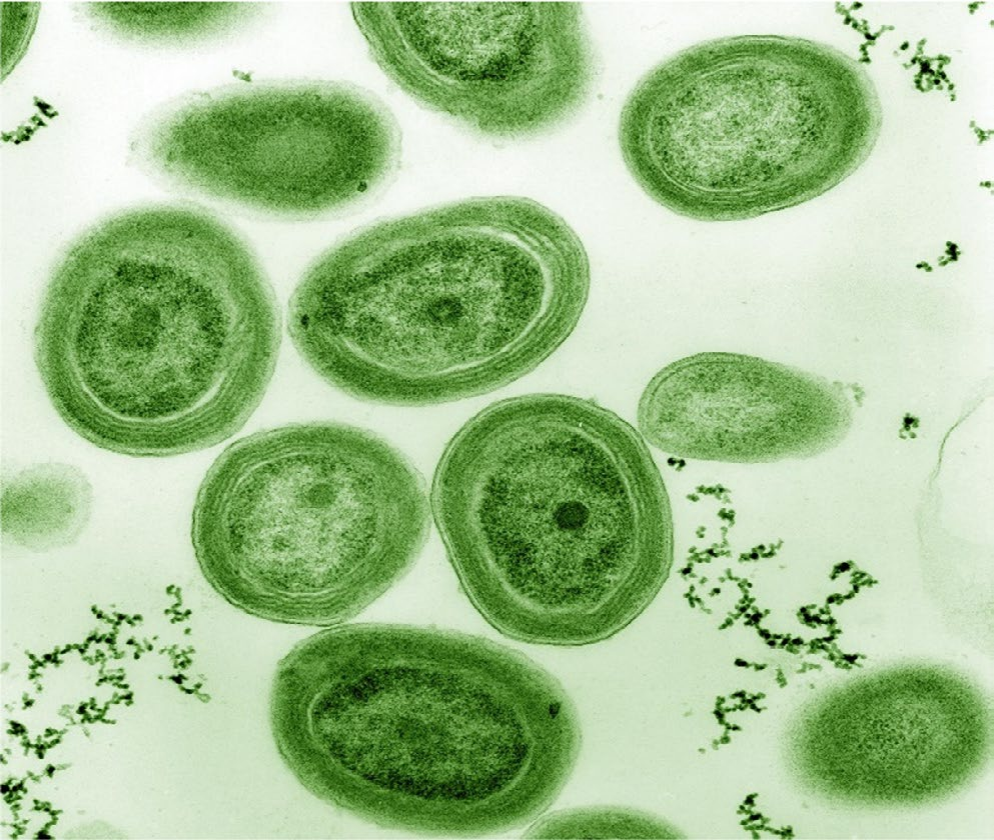
Cells of marine *Prochlorococcus*. These are very small with a diameter of about 0.6 µ. They exist in very large numbers in many of the oceans throughout the world. Surprisingly they were only discovered less than 40 years ago. They are believed to be responsible for much of the oxygen in the atmosphere today. From Wikipedia, in the public domain

The breakthrough is that another large source of oxygen in the ocean has recently been discovered. The source is the tiny microorganism, marine *Prochlorococcus* (Figure 3) which, because of its abundance, is believed by some investigators to produces even more oxygen than cyanobacteria. Enormous amounts of this previously unstudied organism have been found in major oceans of the world including the Pacific, Atlantic, and Southern oceans.

Major contributions have been made in this area by Chisholm, Olson, and others from MIT (Chisholm et al., 1988). They enterprisingly setup equipment for flow cytometry on an ocean‐going vessel. In this technique, fluid containing the cells of interest flows through a narrow capillary tube, and each cell is illuminated by a powerful narrow laser beam as it passes through the tube. The reflected light is analyzed and gives much information. Based on these studies and those of others, a large series of discoveries has been reported.

It is now believed that *Prochlorococcus* is the most abundant photosynthetic organism in the oceans today. Large numbers have been found in the Pacific, Atlantic, and Southern oceans, particularly in the sub‐tropical regions. The organism is extremely small with a diameter of 0.4 to 0.6 microns. This means it is near the limit of resolution of the light microscope, although it gives a signal in flow cytometry. The total number of organisms is gigantic, being estimated at 3 X 10^27^. Sea water from the regions with *Prochlorococcus* typically has a concentration of more than 10^5^ cells per ml. Because of the prodigious number of cells, the total surface area of the cells in the oceans is astronomical.

The photosynthetic properties of the cells that enable them to harvest light are due to transmembrane divinyl chlorophyll alpha and beta complexes. Chlorophyll exists in alpha and beta forms. The alpha version does most of the transfer of energy from light, whereas chlorophyll beta assists at some wavelengths. Vinyl or ethanyl is another component of chlorophyll. Divinyl is unique to *Prochlorococcus* the common form being monovinyl. The chlorophyll of *Prochlorococcus* is thought to be exceptionally efficient at harvesting light (Ulloa et al., 2021).

To summarize this section, the recent discovery of the marine *Prochlorococcus* has identified an enormous new source of oxygen on planet Earth. The implications are not yet fully understood but must be substantial.

Finally, the evolution of oxygen‐producing organisms on our planet Earth invites some interesting speculations. The oxygen molecule is highly reactive and is a component of many food sources. We can assume that we and the other animals that evolved on Earth have taken advantage of the special features of this molecule that readily reacts with so many others. It will be interesting to see whether other forms of life that surely will be discovered on some of the trillions of exoplanets in the universe will be different because they lacked the advantage of oxygen when they developed.

John B West is responsible for the whole of this manuscript.

## CONFLICT OF INTEREST

There is no conflict of interest.
